# Anti-Obesity Effects Evaluation of a Blackcurrant Leaf Standardized Hydro-Alcoholic Extract in Wistar Rat Subjected to a High-Fat Diet

**DOI:** 10.3390/biology13120999

**Published:** 2024-12-01

**Authors:** Gwendoline Bréger, Agnès André, César Cotte, Abderrahim Hammaidi, Aline Amérand, Claude Faivre, Lionel Martignat, Mohamed Yassine Mallem

**Affiliations:** 1 Oniris, Nutrition, Pathophysiology and Pharmacology (NP3), 101 route de Gachet, 44307 Nantes CEDEX 3, France; agnes.andre@oniris-nantes.fr (A.A.); lionel.martignat@oniris-nantes.fr (L.M.); 2 Wamine Industrie, 1 ZI duTaillis, Champtoceaux, 49270 Orée d’Anjou, Francec.faivre@wamine.com (C.F.); 3 Naturopôle Nutrition Santé, PiLeJe Industrie, Les Tiolans, 03220 Saint Bonnet-de-Rochefort, France; c.cotte@pileje-industrie.com; 4 Orphy EA 4324, University of Brest, 6 avenue Victor Le Gorgeu, 29238 Brest, France; aline.amerand@univ-brest.fr

**Keywords:** blackcurrant (*Ribes nigrum*), quercetin, polyphenols, obesity, rat

## Abstract

Blackcurrant (BC) extract was reported to exert anti-obesity effects. However, it is unknown whether BC extract with a composition close to the totum differentially affects obesity when compared to one of its active compounds. We evaluated the anti-obesity effects of a BC standardized hydro-alcoholic leaf extract (BC-HLE) in an HFD-induced obesity rat model and compared them with quercetin (QUE). Thirty-six 12-week-old Wistar rats were divided into six groups: control, HFD, BC-HLE- (41 and 50 mg/kg) and QUE- (0.9 and 50 mg/kg) supplemented HFD rats for 12 weeks. HFD rats developed a moderate obesity, associated with a gut dysbiosis and a change in their total antioxidant capacity. The increase in body weight gain was prevented only by the low dose of BC-HLE and the high dose of QUE. The impaired glucose tolerance by HFD was attenuated by the low dose of QUE. Hepatic glutathione peroxidase activity was increased in the HFD group and only BC-HLE supplementation counteracted this change. The low BC-HLE dose tended to reduce the HFD-induced gut dysbiosis. These findings suggest that while QUE has beneficial effects on obesity-related disorders, the BC-HLE may offer even greater overall benefits and could contribute to preventing obesity and related conditions.

## Simple Summary

This study investigated the potential anti-obesity benefits of blackcurrant extract, specifically its hydro-alcoholic leaf extract, in comparison to quercetin, one of its main active compounds. The rats fed with a high-fat diet developed a moderate obesity, gut imbalance, and changes in antioxidant capacity. Over 12 weeks, rats were given either blackcurrant extract or quercetin in two different doses. The results show that the extract and quercetin supplementations prevented weight gain and partially improved glucose tolerance and antioxidant activity disrupted by the high-fat diet. Importantly, the extract also helped to improve gut health, which was negatively impacted by the diet. These findings suggest that the extract may offer broader health benefits than quercetin alone, potentially making it valuable for preventing obesity and its related health issues. This study is promising, as it highlights the potential of the blackcurrant leaf extract in developing natural approaches to manage and prevent obesity, which could be beneficial for overall public health.

## 1. Introduction

Obesity represents a paramount public health concern that contributes to the onset of a myriad of metabolic and cardiovascular disorders [1]. Extensive research has indicated that the risk of obesity correlates, in part, with the heightened intake of high-fat diet (HFD) [2,3]. Furthermore, HFD have been associated with various complications, such as non-alcoholic fatty liver disease, insulin resistance and certain malignancies [4,5]. The interrelation between obesity and HFD is associated with elevated morbidity rates and a significant reduction in life expectancy [1], highlighting the urgent need for effective prevention and intervention strategies to address this growing crisis.

Dietary supplements are of great interest for addressing metabolic issues related to obesity because they have low toxicity and possible health advantages [6]. Epidemiological studies have indicated that consumption of fruits and vegetables is linked to a lower risk of obesity and chronic diseases [7,8]. Their health benefits are mainly due to high levels of polyphenols, especially anthocyanins [9,10]. Blackcurrant (BC, *Ribes nigrum*) is an important source of various bioactive compounds such as anthocyanins, vitamin C, and γ-linolenic acid. Many studies have indicated the possible protective role of BC (leaf and fruit) against metabolic syndrome, showing improvements in dyslipidemia, hypertension, glucose metabolism, insulin resistance (IR), oxidative status, gut microbiota composition, and obesity [11,12,13,14].

While most research mainly focused on BC fruit, it is essential to highlight that the leaves contain a higher concentration of polyphenolic compounds [11,15]. The latter have a strong correlation with antioxidant capacity, making them more crucial than those found in buds and berries [15]. One of the main active components of BC is quercetin (QUE), which is mostly present in conjugated form with sugar residues. However, in experimental studies and research trials involving dietary supplements, the aglycone variant is typically employed [16]. QUE is widely recognized for its remarkable advantages in combating obesity and related metabolic issues, including its impressive antioxidant properties and its ability to enhance glucose tolerance and improve lipid profiles [17,18,19,20].

The present study was designed to determine the ability of a standardized hydroethanolic leaf extract of blackcurrant (BC-HLE) in preventing the metabolic and functional modifications, including gut microbiota change associated with obesity, in a rat model of diet-induced obesity. To the best of our knowledge, few or no studies have evaluated the effects of a standardized BC-HLE, with a composition close to the totum in the Wistar rat model of obesity induced by an HFD. Moreover, whether the potential beneficial effect of standardized BC-HLE may involve synergistic interaction or multifactorial effects between QUE and other ingredients present in BC remains unknown so far. We hypothesized that long-term supplementation of obese Wistar rat with standardized BC-HLE would produce a greater beneficial effect than that produced by purified QUE, at two doses, the low dose being the level of QUE present in the BC-HLE.

## 2. Material and Methods

### 2.1. Blackcurrant Extract

The fresh leaves of Blackcurrant (*Ribes nigrum* L.) from organic farming were harvested, before flowering, in June 2020 in France (Drôme) and frozen 24 h maximum after the harvest to maximize optimal active content (polyphenols). The blackcurrant extract was produced according to the PhytoStandard^®^ patented extraction process (Institute of vegetal substance, 2002, WO 01/56584A1 patent): the leaves were cold mashed and immediately extracted by hydro-alcoholic leaching. After alcohol evaporation under vacuum and at low temperature the extract was freeze dried. This process allowed for the extraction of compounds from blackcurrant leaves while preserving their integrity and concentrating them; it also enabled us to restore, in an extract, a totum of active ingredients closer to that of the fresh plant, reflecting its therapeutic activity [21]. Blackcurrant leaves compounds are concentrated in the extract. The BC-HLE was analyzed by HPTLC (high-performance thin-layer chromatography) adapting European pharmacopeia monograph (07/2013:2528). The HPTLC analysis of the ethanolic extracts of BC-HLE showed the presence of rutoside and isoquercitroside (Figure S1 in Supplementary Materials). The BC-HLE was also analyzed by HPLC (high-performance liquid chromatography) to assay the amount of isoquercitroside (1.9%), quercetin (2.0%) and proanthocyanin (1.2%). The extract is an isoquercitroside-standardized HLE of blackcurrant (EPS Cassis; according to PiLeJe Laboratoire, Saint Bonnet-de-Rochefort, France).

### 2.2. Experimental Animals

Thirty-six ten-week-old male Wistar rats were procured from Janvier Labs (Le Genest St Isle, France). All the experiments were conducted in accordance with the guidelines from the ethical committee of Pays de la Loire (Ministry authorization, Paris, France, Apafis n° 34339). Animals were housed by two in cages with 12 h light–dark cycles. Rats were acclimatized before the experimental procedures for two weeks and were allowed free access to standard rat diet (3430, Kliba Nafag, Kaiseraugst, Switzerland) and water.

### 2.3. Experimental Procedure

Six groups of six rats were randomly assigned (JavaScript^®^) to different diets for 12 weeks (the experimental unit is the individual animal): Standard, HFD (45% fat, D12451, Research Diets), HFD + BC-HLE (41 or 50 mg dry matter/kg) and HFD + QUE (0.9 or 50 mg/kg, Sigma-Aldrich, St. Louis, MO, USA #337951).

Body weight and food and drink consumption were recorded weekly. At the end of the experimental period, the animals were fasted overnight, anesthetized by an intra-peritoneal injection of a mixture ketamine (60 mg/kg)/medetomidine (120 µg/kg). Blood was collected by cardiac puncture into EDTA and heparin tubes. After centrifugation at 5000× *g* for 15 min at 4 °C, plasma was collected and stored at −80 °C. Liver, visceral adipose tissue and intestine were dissected immediately following blood collection, washed with Krebs solution, frozen in liquid nitrogen, and stored at −80 °C. The adiposity index was calculated from the sum of the individual masses of the epididymal, renal and visceral fat layers, using the following formula: (epididymal fat + renal fat + visceral fat) × 100/final body mass.

### 2.4. Biochemical Parameters

#### 2.4.1. Oral Glucose Tolerance Test (OGTT)

At the beginning of the study and at the 12th week, rats were feed deprived for 4 h before oral gavage with a glucose solution (0.5 g of glucose/mL and 2 g of glucose/kg). Different blood samples were taken at 0, 15, 30, 45, 60, 90 and 120 min after glucose administration. Glycaemia was determined using a glucometer (STAT STRIP Xpress). The OGTT measures the ability of varied organs to take up glucose, which is the body’s main supply of energy.

#### 2.4.2. Determination of Plasma Insulin, Adiponectin and Leptin

Insulin, adiponectin and leptin were measured using commercial ELISA kit supplied by Mercodia (Uppsala, Sweden) for insulin and R&D System (Minneapolis, MN, USA) for adiponectin and leptin. All procedures were followed according to manufacturer’s guidelines. The absorbance was read at 450 nm on Biotek 800 TS absorbance reader (Norgen Biotek, Thorold, Ontario, Canada).

#### 2.4.3. Lipid Profile Analysis in Rat Plasma

On the heparin plasma, total cholesterol (TC), high-density lipoprotein cholesterol (HDL-C) and triacylglycerols (TG) were determined by enzymatic analysis using commercial assay kits (Roche Diagnostics, Basel, Switzerland). Non-esterified fatty acids (NEFA) were determined using a Randox assay kit (Crumlin, UK). All procedures were followed according to manufacturer’s guidelines. Plasma low-density lipoprotein cholesterol (LDL-C) was estimated using Friedewald’s equation: LDL-C = [TC − (HDL-C − (TG/2,2))] mmol/L.

#### 2.4.4. Oxidant Parameters

On the liver and the visceral adipose tissue, the enzyme activities were determined by UV spectrophotometry (UVIKON XL model) at 37 °C. Samples from frozen tissues were placed in an extraction buffer (75 mM Tris and 5 mM EDTA) at 4 °C and pH 7.4 for homogenization with a Polytron homogenizer. After centrifugation at 12,000× *g* for 10 min at 4 °C, the enzymatic activities of superoxide dismutase (SOD), catalase (CAT), glutathione peroxidase (GPx), and the total protein content were determined on the resulting supernatant by ORPHY (Brest university).

#### 2.4.5. Short-Chain Fatty Acid Determination

Short-chain fatty acids (SCFAs), including acetate, propionate, butyrate, isobutyrate, and valerate, were quantified from ≈100 mg of fecal samples by gas chromatography–mass spectrometry as described previously [22].

#### 2.4.6. Microbiota

##### DNA Extraction

DNA was extracted, from the fecal samples, using the ZymoBIOMICS™ 96 MagBead DNA Kit (Zymo Research Corp., Irvine, CA, USA) following a protocol with dual cell lysis (mechanical and chemical). DNA isolation was carried out on a KingFisher Flex automated station (ThermoFisher Scientific Inc., Waltham, MA, USA) according to the manufacturer’s instructions. DNA was quantified by fluorimetry using a Qubit 3.0 (Thermo Fisher Scientific, Waltham, MA, USA). 

##### 16S Metabarcoding Analysis—Library Preparation and Sequencing

The V3–V4 region of the gene encoding 16S ribosomal RNA was amplified by polymerase chain reaction (PCR) using primers 341F and 785R [23]. The amplicons were cleaned up using magnetic AMPure XP beads (Beckman Coulter, Villepinte, France) before adding dual indices and sequencing adapters using the Illumina Nextera XT Index kit (Illumina, San Diego, CA, USA). Each library was cleaned up and quantified by fluorimetry (Qubit^®^ 2.0 Fluorometer), normalized and pooled. The pooled library was denatured before sequencing (2 × 250 paired-end, v2 chemistry) using an Illumina MiSeq (Illumina, San Diego, CA, USA).

##### 16S Metabarcoding Analysis—Data Processing

The sequences were analyzed using a bioinformatic pipeline developed by Biofortis based on Dadaist2 software [24]. Basically, after demultiplexing the barcoded Illumina paired reads, single read sequences were paired for each sample into longer fragments and cleaned. After quality filtering and sequencing error modelling, amplicons variants sequences (ASV) were obtained. A taxonomic assignment of these ASV was performed in order to determine bacterial community profiles.

### 2.5. Statistical Analysis

Data are presented as mean ± SEM. The statistical significance was evaluated by one- or two-way analysis of variance (ANOVA followed by Dunnett’s (one way) or Šídák’s (two ways) multiple comparisons test or Kruskal–Wallis test (one way) when the gaussian distribution were not confirmed followed by Dunn’s multiple comparison tests), Student *t*-test (or Mann–Whitney test when the normality and variance were not confirmed) or PERMANOVA (permutational multivariate analysis of variance) using Bray–Curtis dissimilarity. All data analyses were performed using GraphPad Prism software (version no. 9.5.1; GraphPad Software, Inc., San Diego, CA, USA). *p* < 0.05 was considered to indicate a statistically significant difference.

## 3. Results

### 3.1. Effect of HFD and Supplementations Consumption on Body Weight, Food/Water Intake, Index Adiposity and Abdominal Circumference

**Figure 1:**
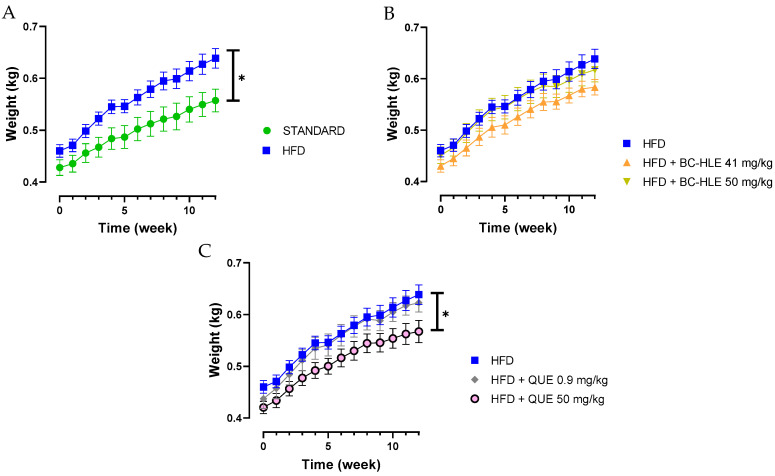
Body weight gain of rats fed experimental diet for 12 weeks. (**A**) Effect of HFD consumption. (**B**) Effect of HFD consumption supplemented with the extract (BC-HLE). (**C**) Effect of HFD consumption supplemented with quercetin (QUE). Values are expressed as mean ± SEM (*n* = 6) and analyzed by using two ways ANOVA (* *p* < 0.05).

Twelve weeks of HFD increased significantly the weekly and final body weight gain (15% compared to final body weight of standard); thus, rats were considered obese (Table 1 and Figure 1A). Despite a lower food intake, the caloric intake and the food efficiency were higher for the HFD group in comparison with standard. The low dose of BC-HLE and the high dose of QUE were the only ones that reduced caloric intake compared to the HFD group (*p* = 0.144 and *p* = 0.012 respectively), but they did not affect food efficiency. Similarly, only the low BC-HLE dose (Figure 1B: *p* = 0.1) and the high QUE dose (Figure 1C: *p* = 0.045) appeared to reduce body weight gain compared to the HFD group. The abdominal circumference and adiposity index of the HFD group increased significantly in comparison to the standard diet. No changes were seen in these measurements among the HFD group receiving supplements.

### 3.2. Effect of HFD and Supplementations Consumption on OGTT

**Figure 2:**
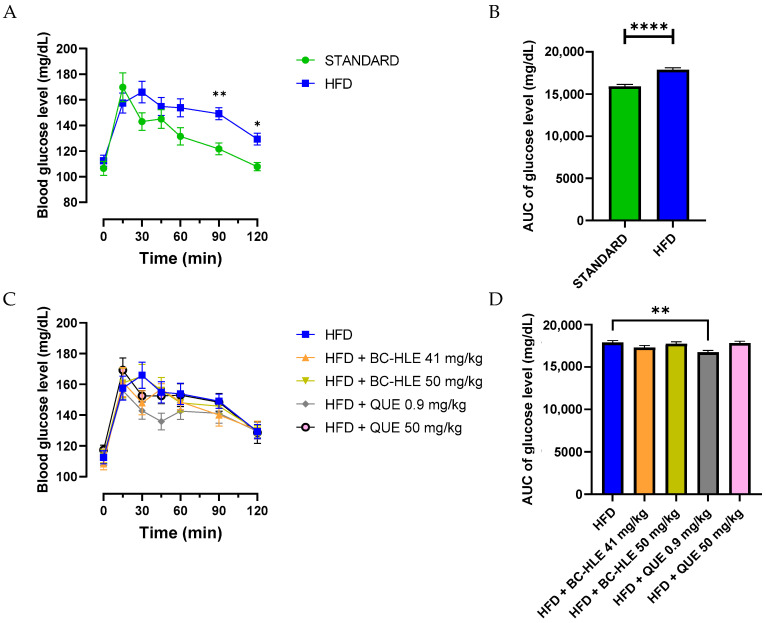
Effect of HFD and supplementations consumption on oral glucose tolerance test (OGTT). (**A–C**) Plasma glucose levels were measured over a 0–120-min period during the OGTT. Aeras under the curves (AUC) for plasma glucose concentrations were calculated from 0 to 120 min during OGTT for the HFD consumption (**B**) and HFD consumption supplemented (**D**). Values are expressed as mean ± SEM (*n* = 6) and analyzed by using two-way ANOVA (A), Student *t*-test (**B**) or one-way ANOVA followed by Dunnett’s multiple comparisons test (**C**) (* *p* < 0.05, ** *p* < 0.01, **** *p* < 0.0001).

Each rat underwent the OGTT to assess insulin sensitivity. The HFD group had a significantly higher glycemia at 90 and 120 min after glucose intake compared to the standard group (Figure 2A). Likewise, the increased area under the HFD glucose curve was significantly different from the standard group (Figure 2B). The lowest doses of supplementation reduced this increase, but only the low quercetin dose showed significant effects (Figure 2C).

### 3.3. Effect of HFD and Supplementations Consumption on Plasma Lipid and Metabolic (Adiponectin, Leptin) Profiles

No differences were observed in plasma triacylglycerols (TG), total cholesterol (TC), high-density lipoprotein cholesterol (HDL-C) and low-density lipoprotein cholesterol (LDL-C) among the groups (Table 2). The HFD group significantly decreased non-esterified fatty acid (NEFA) concentration in comparison with standard. Only the high QUE supplementation tended to counteract this phenomenon (*p* = 0.074).

**Figure 3:**
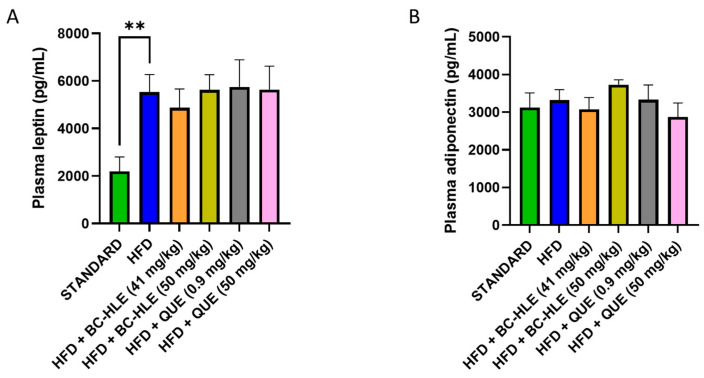
Effect of HFD and supplementations consumption on plasma leptin (**A**) and adiponectin concentration (**B**). Values are expressed as mean ± SEM (*n* = 6) and analyzed by using Student *t*-test (Standard vs. HFD) or ANOVA one-way followed by Dunnett’s multiple comparisons test (** *p* < 0.01).

Plasma concentration of adiponectin and leptin are shown in Figure 3. There was no significant difference in plasma adiponectin concentration among all groups. As expected, plasma leptin concentration significantly increased after 12 weeks of HFD. No significant changes were observed between HFD and HFD with supplementation.

### 3.4. Effect of HFD and Supplementations Consumption on Visceral Adipose Tissue: Protein Expression and Oxidative Profile

**Figure 4:**
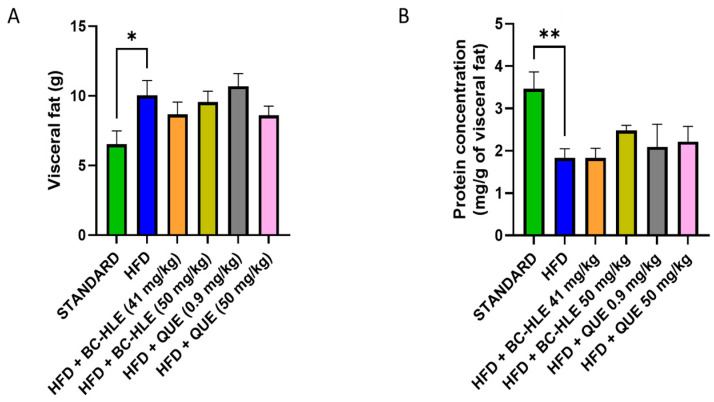
Effect of HFD and supplementations consumption on visceral adipose tissue expansion. (**A**) Weight of visceral fat. (**B**) Protein concentration in visceral fat. Values are expressed as mean ± SEM (*n* = 6) and analyzed by using Student *t*-test (Standard vs. HFD) or Kruskal–Wallis test followed by Dunn’s multiple comparisons test (* *p* < 0.05, ** *p* < 0.01).

**Figure 5:**
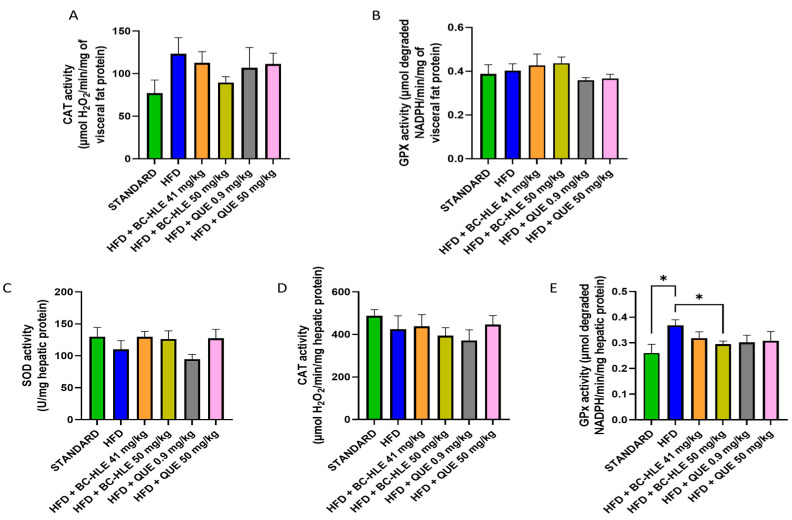
Effect of HFD and supplementations consumption on antioxidant enzyme activities. (**A**) Catalase (CAT) activity in visceral fat. (**B**) Glutathione peroxidase (GPx) activity in visceral fat. (**C**) Superoxide dismutase (SOD) activity in liver. (**D**) CAT activity in liver (**E**) GPx activity in liver. Values are expressed as mean ± SEM (*n* = 6) and analyzed by using Student *t*-test (Standard vs. HFD) or one-way ANOVA followed by Dunnett’s multiple comparisons test (* *p* < 0.05).

The weight of visceral fat and protein concentration in visceral adipose tissue are shown in Figure 4. HFD is characterized by the increase in visceral fat and the decrease in proteins in this tissue after 12 weeks of diet. No significant changes were observed in visceral fat weight by the supplementations. Although, a slight decrease, by the low BC-HLE dose and the high QUE dose, was suggested. The antioxidant status was not significantly affected by HFD and supplementations compared to the standard group (Figure 5A,B). Nevertheless, catalase activity appeared to increase in the HFD group compared to the standard group, although the difference was not statistically significant (*p* = 0.089). Interestingly, a high dose of BC-HLE seemed to reduce this activity, but the effect did not reach a significant level (*p* = 0.185).

### 3.5. Effect of HFD and Supplementations Consumption on Hepatic Tissue: Antioxidant Profile

As shown in Figure 5C–E, the liver antioxidant status was altered by HFD. The SOD and CAT activities were slightly reduced. No significant difference was noticed by supplemented HFD groups. Concerning SOD activity, supplementations, except the low QUE dose, seemed to increase its activity compared to HFD group. HFD clearly raised the GPx activity in comparison with standard. All supplementations had minimized it, but it was only significant for the high BC-HLE dose.

### 3.6. Effect of HFD and Supplementations Consumption on Microbiota and Its Metabolites

The impact on gut microbiota composition after 12 weeks of HFD and HFD with supplementations was evaluated using 16S rRNA sequencing. All 36 rats provided a stool sample deemed quantitatively sufficient for taxonomic analyses up to genus identifications analysis.

**Figure 6:**
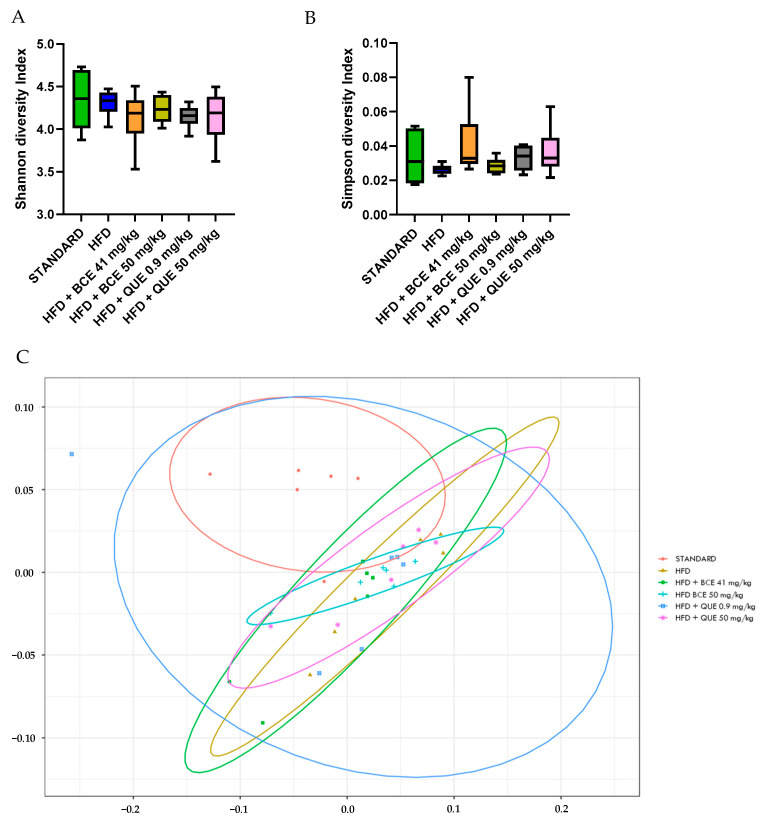
Effect of HFD and supplementations consumption on α-diversity (**A**,**B**) and β-diversity (**C**). Values are analyzed by using Student *t*-test (Standard vs. HFD), one-way ANOVA or Kruskal–Wallis test (**A**,**B**) and PERMANOVA Bray (**C**) (*n* = 6).

After 12 weeks of HFD diet, there was no significant change in α-diversity, nor was there with supplementations (Figure 6A,B). Furthermore, a permutational multivariate analysis of variance (PERMANOVA) using Bray–Curtis dissimilarity was performed to evaluate β-diversity of the gut microbiome. In Figure 6C, all the clusters of the different groups are represented at the phylum level (*p* = 0.099). Further analyses at the family and genus level were assessed between all groups. The HFD group showed a clear difference with standard cluster (*p* = 0.01 at the phylum, family and genus level). The HFD group supplemented with BC-HLE formed a different cluster than the HFD group alone (*p* = 0.02 at the family level and *p* = 0.01 at the genus level). The comparison of the HFD group according to the dosages of BC-HLE revealed a significant difference between clusters only with the highest dose (*p* = 0.03 at the family level). Concerning the QUE supplementations, just the low dose formed a large cluster significantly different with the HFD group (*p* = 0.03 at the genus level).

**Figure 7:**
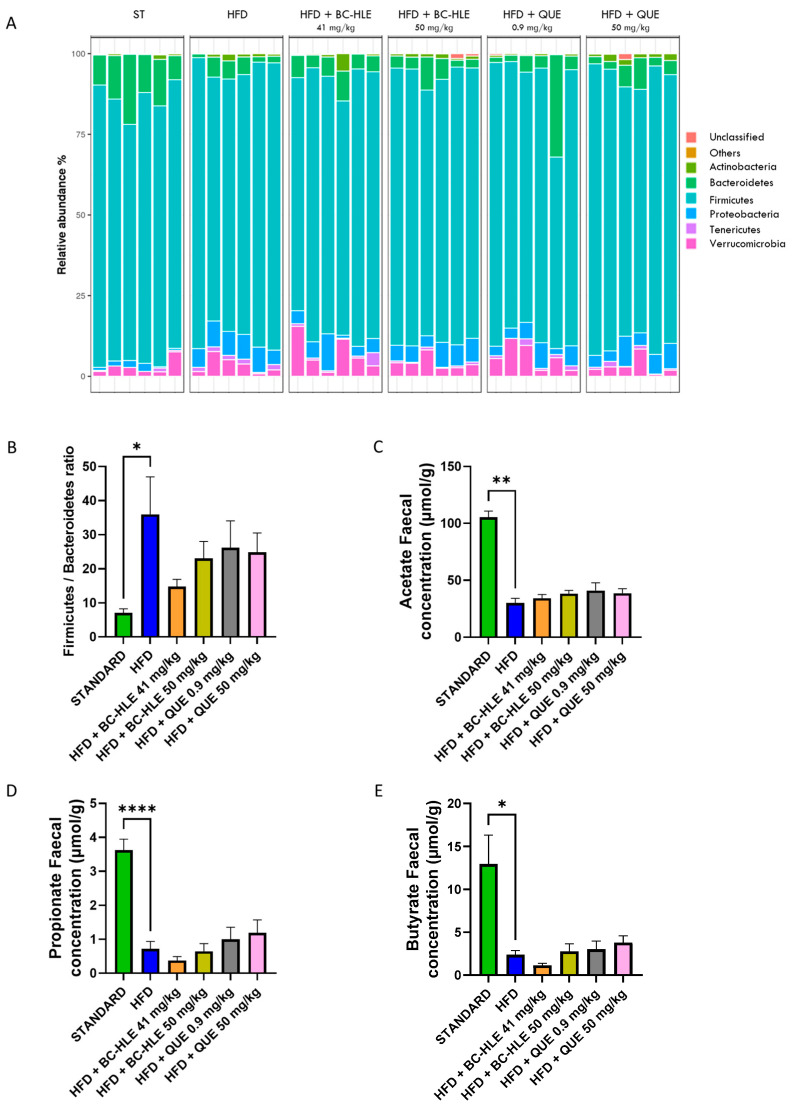
Effect of HFD and supplementations consumption on microbiota (Phylum level) and its metabolite. (**A**) Relative abundance (%) at the phylum level after 12 weeks of experimental diet. (**B**) *Firmicutes*/*Bacteroidetes* ratio. (**C**–**E**) Short-chain fatty acid concentration in stool. Values are expressed as mean ± SEM (*n* = 6) and analyzed by using Student *t*-test or Mann–Whitney test (Standard vs. HFD) or one-way ANOVA followed by Dunnett’s multiple comparisons test (* *p* < 0.05, ** *p* < 0.01, **** *p* < 0.0001).

To investigate the specific microbiota changes through HFD and HFD with supplementations, we analyzed the taxonomic composition in different groups, as shown in Figure 7A,B). At the phylum level, the relative abundances of *Bacteroidetes* (*p* = 0.002) decreased, while *Proteobacteria* (*p* < 0.001) and *Tenericutes* (*p* = 0.009) increased in the HFD group compared to standard. The gut microbiota of the HFD group was characterized by a rise in the *Firmicutes/Bacteroidetes* ratio. Only trends were observed for HFD with supplementation compared with the HFD group, except for the high BC-HLE dose which significantly decreased the relative abundance of *Tenericutes* (*p* = 0.026). However, it is noteworthy that the low BC-HLE dose slightly reduced the *Firmicutes/Bacteroidetes* ratio (*p* = 0.09).

Twelve weeks of HFD lowered acetate, propionate and butyrate concentration in feces (Figure 7C–E). Supplementations had no significant effects on acetate and propionate concentration compared to HFD group. The reduction in butyrate concentration observed in the HFD group appeared to be further exacerbated by the administration of the low BC-HLE dose, though this effect was not statistically significant (*p* = 0.279). Conversely, only the high dose of QUE demonstrated a tendency to increase butyrate levels, albeit without reaching statistical significance (*p* = 0.344).

## 4. Discussion

In this study, we investigated the potential anti-obesity effects of a standardized hydro-alcoholic leaf extract of blackcurrant (BC-HLE) at doses of 41 and 50 mg/kg in a rat model of HFD-induced obesity. We also compared its effects to QUE administered at two different doses. The HFD, containing 45% fat from lard, led to moderate obesity, evidenced by a 15% increase in final body weight compared to rats on a standard diet [25]. This obesity was further characterized by increased abdominal circumference, visceral fat accumulation, and a higher adiposity index, as well as decreased glucose tolerance and alterations in total antioxidant capacity. Notably, the HFD-fed rats in our study did not exhibit IR, as indicated by an unchanged leptin/adiponectin *ratio* and HOMA-IR, both established markers of IR [26]. Additionally, there were no alterations in lipid profile or increases in circulating NEFA levels, which are known as contributors to the onset and progression of IR and disruptions in lipid metabolism [27,28]. These findings contrast with previous studies reporting that fat-enriched diets often lead to marked obesity-related conditions, including hypertriglyceridemia and IR [29]. However, this result supports the notion that the moderate level of obesity induced by our HFD was likely insufficient to produce significant disturbances in glucose homeostasis.

The dosage of BC-HLE was selected based on its traditional use in dogs for antioxidant and anti-inflammatory purposes—the primary target species for such supplementation—and was adjusted for rats using the human equivalent dose (HED) calculation method [30], resulting in doses of 41 to 50 mg dry matter/kg. Although the European Medicines Agency (EMA/HMPC/745353/2016) could not identify specific blackcurrant constituents with recognized therapeutic activity, numerous studies have documented QUE as one of the main flavonoids in BC, associated with various health benefits [11,18]. We, therefore, chose to compare the anti-obesity effects of BC-HLE with those of QUE. The lower QUE dose (0.9 mg/kg) corresponded to its natural occurrence in 41 mg/kg of BC-HLE, while the higher dose (50 mg/kg) was exploratory, based on studies showing anti-obesity and anti-diabetic effects at comparable doses [20,31]. Our BC-HLE was standardized according to European Pharmacopoeia guidelines to ensure that it closely resembled the natural composition of the leaf. This study aimed to determine whether the effects of BC-HLE are primarily due to QUE at its natural level and explore the benefits of higher QUE dose.

We found that the low BC-HLE dose supplementation reduced weight gain, although not significantly, unlike the high QUE dose. It is noteworthy that BC-HLE’s effect on weight gain was not dose-dependent; high dose did not further reduce weight or visceral fat. The reason to account for the lack of dose-dependency in the BC-HLE effect is not readily apparent. One possibility is that the amount of simple sugars present in the high BC-HLE dose could have contributed to limiting its benefit on weight gain, as caloric intake was found to be increased. However, this hypothesis is unlikely, as caloric intake was also increased in rats fed low QUE dose. The complex interaction between the bioactive constituents of BC-HLE that may occur with the dose increase may be another possibility to explain the lack of dose-dependency. This emphasizes the need to better understand the relationship between BC-HLE phytochemicals and their mode of action. Indeed, until now, it was unclear whether the beneficial effect of BC-HLE may involve synergistic interaction or multifactorial effects between QUE and other phytochemicals present in BC-HLE. Additionally, the low QUE dose failed to prevent weight gain compared to the low BC-HLE dose, suggesting a likely synergistic effect of BC-HLE compounds in preventing obesity. In agreement with this suggestion, several compounds found in BC leaves such as kaempferol and hydroxycinnamic acid derivatives have been described for their ability to manage obesity and its related alterations [32,33]. The inefficacy of the low QUE dose might be due to its low bioavailability in its aglycone form, which is less absorbed in rat intestine [34]. Nonetheless, the high QUE dose prevented weight gain, indicating that the anti-obesity effect of QUE, depends more on the dose level than the chemical form. Interestingly, the high QUE dose was almost as effective as the low BC-HLE dose, suggesting that a 50 times larger dose of QUE might be required to achieve the same benefit as BC-HLE.

A strong link between polyphenol-rich fruit or vegetable extracts and improved insulin sensitivity or glucose homeostasis has been well documented [35,36,37]. Although HFD did not lead to any significant changes in the leptin/adiponectin *ratio* or the HOMA-IR in our study, it resulted in a decrease in insulin sensitivity. We found that only the low QUE dose and the low BC-HLE dose slightly improved glucose tolerance hampered by HFD, suggesting that BC-HLE, through its flavonoid quercetin, can positively but modestly modulate glucose uptake. This effect is likely not due to the low dose used, as higher dose did not change the AUC of glucose levels. It is reasonable to assume that there may be antagonistic effects due to phytochemicals present in the BC-HLE, which become effective under some threshold dose, and that QUE could exert opposite effects on glycaemia that depend on the dose level, which refers to the ambivalent character of QUE, as described elsewhere [38,39]. Interestingly, despite the high QUE dose supplementation reduced weight gain, it did not improve glucose tolerance as expected. The lack of this parallel change on body weight and OGTT is unclear but could be due to a possible unfavorable direct effect on the mechanisms of the glucose peripheral use. Improvement in glucose homeostasis may occur at higher doses than those used in the present study, as higher BC dosages (>100 mg/kg) have shown their ability to alleviate IR and to improve glucose homeostasis [12,13,14]. However, studies had tested parts of the BC other than the leaf, using water or methanolic extracts. Thus, care must be taken not to extrapolate the present results to other works using other kinds of BC extracts or non-leaf BC extracts at considerably higher doses.

Obesity induced by HFD consumption is largely documented to be associated with oxidative stress, which contributes to the development of obesity-related metabolic and endocrine disorders [40,41]. We found that HFD increased CAT activity in visceral fat and GPx activity in liver but decreased hepatic SOD activity, albeit without consistently reaching statistical significance. These findings seem to disagree with the global down-regulation process in oxidative defenses as previously reported under obesity condition [40,42,43,44], but suggest the establishment of a global antioxidant capacity that has likely been developed to counteract the HFD-induced obesity alterations. Supplementations tended to normalize the observed variations, but without a noticeable increase in the antioxidant abilities in the examined tissues, when compared to the control group, which is consistent with previous studies [45,46]. Furthermore, an opposite effect appears to occur between the different supplementations, which is probably related to the ambivalent nature of QUE [39] or polyphenols [47]. Whether such a special effect may be associated with the protective effect of BC-HLE or QUE remains speculative and needs to be elucidated.

Available research evidence supports a link between the gut microbiome and obesity [48], as well as BC’s ability to modulate gut microbiota composition [12,49]. Thus, we explored the gut microbial changes in Wistar rats fed an HFD with or without BC-HLE or QUE supplements. After 3 months, HFD-feeding altered the gut microbiome, illustrated by changes in abundance of the main bacterial Phyla and decrease in SCFAs, reflecting a great effect of dietary fat on the gut flora in the HFD group. We found that HFD-fed rats strongly exhibited an increase in the *Firmicutes*/*Bacteroidetes* ratio, supposedly linked to obesity [50]. Contrary to previous studies showing a positive effect of BC or QUE upon gut flora [12,49,51], our supplementations were unable to significantly modify the *Firmicutes*/*Bacteroidetes* ratio, arguing against the hypothesis that the alleviation of gut dysbiosis is a necessary prerequisite to allow the BC-HLE or QUE to produce their beneficial effects. However, although not significant, only the low BC-HLE dose tended to restore the *Firmicutes*/*Bacteroidetes* ratio towards standard levels. Thus, it is highly probable that prevention of weight gain and the improvement of glucose tolerance observed in the current study might involve gut microbiota-dependent mechanisms that warrants further investigation.

Besides impairment in the gut microbiota composition, HFD-feeding strongly decreased the production of fecal SCFAs, which is consistent with other studies [20,52,53]. SCFAs are known to have protective effects against obesity [54,55,56,57]. Thus, the SCFA decrease observed may be considered an indicator of the development of obesity and its related disorders. It should be stated, however, that SCFA levels in feces are not a direct measure of SCFA production in the gut, but rather a result of SCFA production after subtracting SCFA absorption. Therefore, it might be hypothesized that higher microbial SCFA production could increase gut SCFA absorption, resulting in lower levels of SCFA excretion in feces. Our results show that BC-HLE as well as QUE supplementations were ineffective at restoring the fecal level of SCFAs at a value close to the control group. However, opposite effects on fecal butyrate concentrations were observed. The low BC-HLE dose lowered butyrate concentration compared to the HFD group, while the high QUE dose enhanced it. Controversial results regarding the effect of BC upon butyrate levels have also been reported in other studies [12,52,58,59,60], and this emphasizes the need for further research to better define the biological significance of the change in butyrate levels following BC or QUE supplementation.

There are some limitations of the current study. Firstly, our small sample size may have limited the ability to detect differences between groups; for instance, the microbiota analysis via 16S RNA is potentially lacking the sufficient power to detect phylum-level differences. A higher number of rats will be needed in future studies to allow a more relevant interpretation of results and to draw a conclusion on the effects of BC-HLE *versus* QUE upon obesity and related disorders. Secondly, the sugar content of BC-HLE was unknown, which is necessary for accurately assessing its impact. Thirdly, our HFD only induced a moderate obesity without substantial metabolic dysregulations, which may have limited the ability to detect more pronounced benefits from the botanical supplementations. Whether the obesity level or the fat/sugar level would differently influence the BC supplementations is still unknown and needs to be thoroughly investigated.

In summary, we showed that long-term supplementation with both BC-HLE and QUE doses impacted obesity parameters in HFD-induced obese Wistar rats differently, particularly in mitigating weight gain, improving glucose tolerance, and modulating oxidative stress. The observed advantage of low BC-HLE dose over a low dose of QUE supports the hypothesis that the potential anti-obesity effect that may involve a role of QUE within the BC-HLE is most likely dependent on its interaction with other BC phytochemicals. More interestingly, we revealed that purified QUE, when used at a 50 times higher dose, induced an effect almost equivalent to that conferred by the low BC-HLE dose. This observation suggests that 0.9 mg/kg does not carry the majority of the BC-HLE effect and strengthens the hypothesis that other phyto-compounds could have acted synergistically to enhance the action of QUE in the BC-HLE group, by mechanisms that remain to be elucidated.

## 5. Conclusions

In conclusion, this study suggests that long-term supplementation with BC-HLE may help prevent obesity and related metabolic disorders. However, further research is needed to better understand the mechanisms underlying the effects of BC-HLE to clarify its apparent dose-dependent inhibitory effect on weight gain and to determine whether this effect is linked to specific changes in the intestinal microbiota.

## Figures and Tables

**Table 1:** Body weight gain, food intake, caloric intake, food efficiency and body mass index of rats fed the experimental diets for 12 weeks (HFD: High-Fat Diet; BC-HLE: Blackcurrant hydroethanolic leaf extract; QUE: quercetin). Food efficiency was calculated as (body weight gain (g)/food intake (g)) × 100. Adiposity index was calculated with the formula: (epididymal fat + renal fat + visceral fat) × 100/final body mass. Values are expressed as mean ± SEM (*n* = 6) and analyzed by Student *t*-test or Mann–Whitney test (Standard vs. HFD), one- or two-ways ANOVA or Kruskal–Wallis test followed by Dunnett’s, Šídák’s or Dunn’s multiple comparison tests (HFD and HFD supplemented); * *p* < 0.05, ** *p* < 0.01, *** *p* < 0.001, **** *p* < 0.0001 for HFD compared to Standard group and # *p* < 0.05 compared to HFD group.

Group	Body Weight Gain (g/12 Weeks)	Food Intake (g/Day)	Caloric Intake (Kcal/Day)	Food Efficiency (%)	Abdominal Circumference (cm)	Adiposity Index
Standard	130 ± 8	27 ± 1	84 ± 3	6	19.4 ± 0.4	3.7 ± 0.3
HFD	178 ± 9 **	20 ± 1 ***	96 ± 5 **	10 ****	21.5 ± 0.4 **	5.2 ± 0.3 *
HFD + BC-HLE (41 mg/kg)	154 ± 8	19 ± 1	89 ± 5	10 ± 1	21.3 ± 0.5	4.7 ± 0.1
HFD + BC-HLE (50 mg/kg)	166 ± 14	20 ± 1	94 ± 6	10 ± 1	21.4 ± 0.3	5.1 ± 0.4
HFD + QUE (0.9 mg/kg)	185 ± 10	20 ± 1	95 ± 6	11 ± 1	21.3 ± 0.3	5.5 ± 0.4
HFD + QUE (50 mg/kg)	147 ± 12	18 ± 1 #	86 ± 6 #	9 ± 1	20.6 ± 0.5	5.3 ± 0.4

**Table 2:** Plasma triacylglycerols (TG), total cholesterol (TC), HDL-cholesterol (HDL-C), LDL-cholesterol (LDL-C) and non-esterified fatty acid (NEFA) level in rats fed the experimental diet. (HFD: High-Fat Diet; BC-HLE: Blackcurrant hydroethanolic leaf extract; QUE: quercetin). Values are expressed as mean ± SEM (*n* = 5–6) and analyzed by Mann–Whitney test (Standard vs. HFD) or Kruskal–Wallis test followed by Dunn’s multiple comparisons test; * *p* < 0.05 HFD compared to Standard group. One rat from HFD + BC-HLE 41 mg/kg and HFD + QUE 0.9 mg/kg group were removed because LDL-C could not be calculated by Friedewald’s equation.

Group	TG (mmol/L)	TC (mmol/L)	HDL-C (mmol/L)	LDL-C (mmol/L)	NEFA (mmol/L)
Standard	0.93 ± 0.11	1.71 ± 0.08	1.16 ± 0.06	0.13 ± 0.04	0.37 ± 0.04
HFD	0.84 ± 0.07	1.61 ± 0.08	1.07 ± 0.08	0.17 ± 0.02	0.21 ± 0.03 *
HFD + BC-HLE (41 mg/kg)	1.19 ± 0.16	1.88 ± 0.17	1.23 ± 0.11	0.14 ± 0.03	0.30 ± 0.03
HFD + BC-HLE (50 mg/kg)	0.79 ± 0.04	1.70 ± 0.11	1.14 ± 0.07	0.20 ± 0.04	0.19 ± 0.04
HFD + QUE (0.9 mg/kg)	0.81 ± 0.09	1.62 ± 0.12	1.11 ± 0.08	0.18 ± 0.05	0.20 ± 0.05
HFD + QUE (50 mg/kg)	0.93 ± 0.09	1.52 ± 0.09	0.98 ± 0.07	0.13 ± 0.03	0.33 ± 0.04

## Data Availability

The original data presented in the study are openly available in Zenodo at DOI: https://doi.org/10.5281/zenodo.13759286 accessed on 13 September 2024.

## Supplementary Materials

The following supporting information can be downloaded at: https://www.mdpi.com/article/10.3390/biology13120999/s1, Figure S1: a: Phytochemical profile of BC-HLE on the right, on the left control solution, b: HPLC-MS profile of BC-HLE.
