# Supplementary material for: Anti-Obesity Effects Evaluation of a Blackcurrant Leaf Standardized Hydro-Alcoholic Extract in Wistar Rat Subjected to a High-Fat Diet
Source: Biology (Basel). 2024 Dec 1;13(12):999. doi: 10.3390/biology13120999 (PMC11727514; doi:10.3390/biology13120999)
Supplement: Supplementary file 1 [file biology-13-00999-s001.zip › biology-3262461-supplementary.pdf]

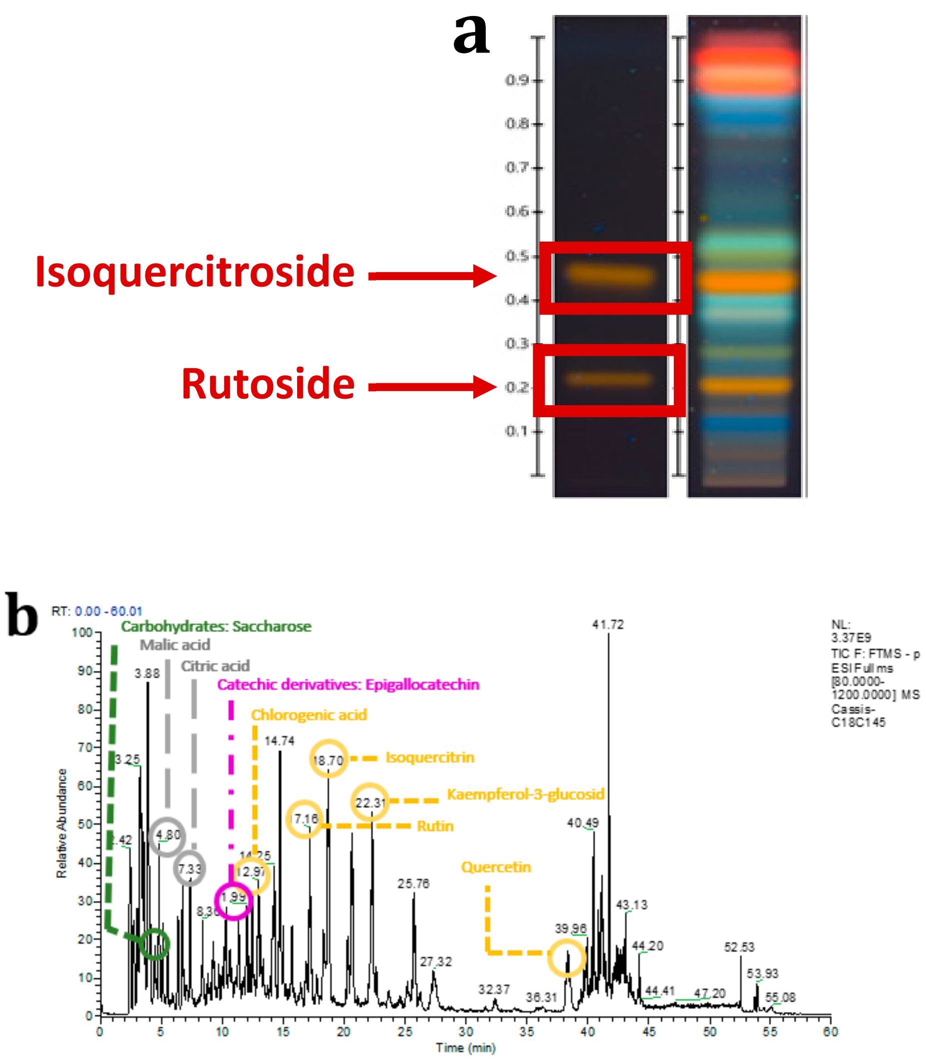

Figure S1:

a: Phytochemical profile of BC-HLE on the right, on the left control solution

b: HPLC-MS profile of BC-HLE
